# Genetic progress in cowpea [*Vigna unguiculata* (L.) Walp.] stemming from breeding modernization efforts at the International Institute of Tropical Agriculture

**DOI:** 10.1002/tpg2.20462

**Published:** 2024-05-22

**Authors:** Patrick Obia Ongom, Christian Fatokun, Abou Togola, Ibnou Dieng, Stella Salvo, Brian Gardunia, Saba Baba Mohammed, Ousmane Boukar

**Affiliations:** ^1^ International Institute of Tropical Agriculture (IITA) Kano Nigeria; ^2^ International Institute of Tropical Agriculture (IITA) Ibadan Nigeria; ^3^ International Maize and Wheat Improvement Center (CIMMYT) Nairobi Kenya; ^4^ Bayer Crop Science Chesterfield Missouri USA

## Abstract

Genetic gain has been proposed as a quantifiable key performance indicator that can be used to monitor breeding programs’ effectiveness. The cowpea breeding program at the International Institute of Tropical Agriculture (IITA) has developed and released improved varieties in 70 countries globally. To quantify the genetic changes to grain yield and related traits, we exploited IITA cowpea historical multi‐environment trials (METs) advanced yield trial (AYT) data from 2010 to 2022. The genetic gain assessment targeted short duration (SD), medium duration (MD), and late duration (LD) breeding pipelines. A linear mixed model was used to calculate the best linear unbiased estimates (BLUE). Regressed BLUE of grain yield by year of genotype origin depicted realized genetic gain of 22.75 kg/ha/year (2.65%), 7.91 kg/ha/year (0.85%), and 22.82 kg/ha/year (2.51%) for SD, MD, and LD, respectively. No significant gain was realized in 100‐seed weight (Hsdwt). We predicted, based on 2022 MET data, that recycling the best genotypes at AYT stage would result in grain yield gain of 37.28 kg/ha/year (SD), 28.00 kg/ha/year (MD), and 34.85 kg/ha/year (LD), and Hsdwt gain of 0.48 g/year (SD), 0.68 g/year (MD), and 0.55 g/year (LD). These results demonstrated a positive genetic gain trend for cowpea, indicating that a yield plateau has not yet been reached and that accelerated gain is expected with the recent integration of genomics in the breeding program. Advances in genomics include the development of the reference genome, genotyping platforms, quantitative trait loci mapping of key traits, and active implementation of molecular breeding.

## Core Ideas

- Historical cowpea yield trial data were exploited to estimate genetic gain realized in the past 12–15 years.
- Realized genetic gain estimates for grain yield depicted positive progress in three key cowpea breeding pipelines.
- Predicted gain estimates portrayed a future increase in the rate of genetic gain in cowpea.
- Accelerated genetic improvement is expected with the recent integration of genomics into the breeding program.

**Abbreviations**
AYT: advanced yield trial
BLUE: best linear unbiased estimates
BLUP: best linear unbiased predictions
KASP: kompetitive allele‐specific polymerase chain reaction
MET: multi‐environment trial
QTL: quantitative trait loci
SNPs: single nucleotide polymorphisms
SSD: single seed descent

## 1 INTRODUCTION

Cowpea [*Vigna unguiculata* (L.) Walp., Fabaceae (2*n*  =  2×  =  22)] is a crucial legume crop in the tropical and subtropical regions of the world and often a principal source of protein and minerals for low‐income people in such regions (Abate et al., 2012; Boukar et al., 2019; Ehlers & Hall, 1997). Millions of people in sub‐Saharan Africa (SSA) depend on cowpea as a key food source and income. Consequently, the world's highest production and consumption of cowpea is in SSA, with the top producers being Nigeria, Niger, and Burkina Faso, which together account for about 60% of the global production (FAOSTAT, 2021). According to the Food and Agriculture Organization, the world production of cowpea in 2021 was estimated to be about 8.99 million metric tons from a harvested area of about 14.91 million ha (FAOSTAT, 2021). In most parts of the world, including Western Africa, the crop is predominantly grown by small‐scale farmers under dryland conditions due to its inherent drought tolerance and atmospheric nitrogen‐fixing ability that enhances soil fertility (Boukar et al., 2019; Horn & Shimelis, 2020; B. B. Singh, 2014; B. B. Singh et al., 1997). Cowpea is an excellent component of the traditional mixed cropping systems in most cowpea growing areas (Horn & Shimelis, 2020). Cowpea is consumed in different forms including grains, green pods, and leaves that are eaten as leafy vegetable, whereas the vines/haulms are used as animal feed (Boukar et al., 2019; Kebede & Bekeko, 2020; B. B. Singh et al., 2003).

Among legumes, cowpea stands out as a potential food and nutritional security crop (da Silva et al., 2018), and this realization has, in recent times, drawn significant research attention to this crop that has been deemed as “the African orphan crop” (Murdock et al., 2013). Despite its potential, cowpea productivity has been limited by several constraints, both biotic and abiotic, particularly diseases, insect pests, parasitic weeds, drought and heat stresses, and soil nutrient deficiencies (Boukar et al., 2019; C. A. Fatokun et al., 2002; Horn & Shimelis, 2020; Nkomo et al., 2021). Yields on SSA farmers’ fields are still low, varying from 100 to 599 kg/ha compared to potential yields of 1500–3000 kg/ha (Boukar et al., 2018; Horn & Shimelis, 2020). At the International Institute of Tropical Agriculture (IITA), there have been tremendous efforts to enhance cowpea productivity through the development of improved lines. These efforts have resulted in releases of 80 IITA supported improved cowpea varieties between 1970 and 2018 in SSA (Maria Figueira Gomes et al., 2019). These varieties are characterized by drought and heat tolerance (Agbicodo et al., 2009; C. A. Fatokun et al., 2012; Hall, 2004; Muchero et al., 2013), striga resistance (Boukar et al., 2018; Omoigui et al., 2010; Omoigui, Kamara, et al., 2017; B. B. Singh et al., 2006; B. B. Singh & Emechebe, 1990), insect resistance (Boukar et al., 2020; Myers et al., 1996; Omoigui, Ekeuro, et al., 2017; Ongom et al., 2022; S. R. Singh, 1977; Togola et al., 2020), high protein and mineral contents (Boukar et al., 2011), high yield potential, and early to medium maturity (Boukar et al., 2019; Kamara et al., 2012; Ongom, Fatokun, Togola, Oyebode, et al., 2021; B. B. Singh, 2007; B. B. Singh & Ntare, 1985). However, with the impending climate change dynamics and population surge, the genetic gain realized from breeding efforts must be accelerated to meet the ever‐increasing global food demands.

Genetic gain has been defined as the increase in performance expected or realized annually through artificial selection (Covarrubias‐Pazaran, 2020; Rutkoski, 2019a, 2019b; Xu et al., 2017). According to Eberhart (1964), when a genetic trend is linear, the rate of genetic gain per year that is realized can be estimated by fitting the least squares regression line of the average breeding value of a trait on the year of genotype creation. Realized genetic gain uses either historical phenotypic data obtained over the years from the evaluation of breeding lines at a specific testing stage as the breeding program evolves or from a set of released varieties evaluated all together in an experiment (era trials). While offering precise estimates of genetic gain, era trials pose logistical challenges (extra costs) and lack real‐time monitoring for timely strategy adjustments (Menkir et al., 2022; Prasanna et al., 2022). The realized gain per year is obtained by regressing the phenotypic data to the year of creation of the germplasm used (Covarrubias‐Pazaran, 2020; Mackay et al., 2011; Rutkoski, 2019a). Estimating the rates of genetic gain using historical data allows for periodic monitoring of the program and has been successfully implemented across various crop breeding programs (Khanna et al., 2022; Menkir et al., 2022; Prasanna et al., 2022; Seck et al., 2023). In contrast, expected genetic gain (also referred to as predicted genetic gain) uses the parameters from breeders’ equation (heritability, selection intensity, and genetic variance) computed for a single season to estimate the response to selection and infer the rate of genetic gain (Falconer & Mackay, 1996; Rutkoski, 2019b; Walsh & Lynch, 2018). Given its relevance in breeding, regular estimation of the rate of genetic gain has been proposed to monitor the effectiveness of breeding programs (Covarrubias‐Pazaran, 2020; Rutkoski, 2019a). Genetic gain estimation has been conducted in several crops (Ayenew et al., 2021; Bezaweletaw et al., 2006; Karmakar & Bhatnagar, 1996; Tadesse et al., 2018; Yadav et al., 2021).

Genetic gain estimation for cowpea cultivars developed in the Sudan Savannas of Nigeria was last conducted in 2011 using data from 1974 to 2004 (Kamara et al., 2011). Since then, there have been tremendous efforts in modernizing breeding programs to accelerate the rate of genetic gain in cowpea, yet the impact has not been assessed. For instance, the cowpea breeding program at IITA has been tapping from recent genomic advances in cowpea (Boukar et al., 2018; Muñoz‐Amatriaín et al., 2017) to develop various molecular breeding platforms. Some of these included developing and deploying markers for parental fingerprinting and verifying true hybridity in F_1_ populations, thus providing quality control and assurance of the program (Ongom, Fatokun, Togola, Salvo, et al., 2021). Molecular markers have also been deployed to understand genetic diversity and structure in germplasm being utilized in the breeding program (C. Fatokun et al., 2018; Gbedevi et al., 2021; Muñoz‐Amatriaín et al., 2021). Genome mapping of key traits has been conducted (Agbicodo et al., 2010; Huynh et al., 2015; Kusi et al., 2018; Ongom et al., 2022), and markers associated with some of these traits, especially aphids and bacterial blight resistances, were deployed in forward breeding. In addition to developing genomic tools, the program adjusted the breeding scheme, moving from traditional pedigree to single seed descent (SSD) for rapid generation advancement, enabling at least three generations per year coupled with molecular screening for seed purity and quality. Concurrently, efforts were made toward improving field uniformity and deploying powerful experimental designs to increase the accuracy of phenotypic data. These practices are key elements of genetic gain; hence, positive changes in these practices are expected to improve the genetic progress of a breeding program over time (Gudi et al., 2022; Xu et al., 2017). The breeding program at IITA has since deployed some of these practices across three breeding pipelines: short duration (SD), medium duration (MD), and late duration (LD). Given the modernization efforts the program introduced in the past 5 years, assessing genetic progress made due to these changes is paramount. The objective of this study was to elucidate the genetic gain made in the cowpea breeding program across three pipelines, utilizing historical yearly data of genetic materials evaluated in advanced yield trials (AYTs).

**Core Ideas**

- Historical cowpea yield trial data were exploited to estimate genetic gain realized in the past 12–15 years.
- Realized genetic gain estimates for grain yield depicted positive progress in three key cowpea breeding pipelines.
- Predicted gain estimates portrayed a future increase in the rate of genetic gain in cowpea.
- Accelerated genetic improvement is expected with the recent integration of genomics into the breeding program.

## 2 MATERIALS AND METHODS

### 2.1 Genetic materials

The genetic materials used in the study were inbred genotypes developed for three breeding pipelines: SD, MD, and LD. These comprised 268, 290, and 251 genotypes in the SD, MD, and LD pipelines, respectively (Table 1). The year of origin of the genotypes (i.e., the year the genotypes became fixed) covered the period from 2006 to 2019 for the SD, 2004 to 2019 for MD, and 2006 to 2018 for LD pipelines. Every year starting from 2010 to 2022, fixed genotypes were tested at AYT, and it is the historical data from the evaluations of these genotypes that were used in the current study to estimate genetic gain.

**TABLE 1:** Number, year of origin, and testing of genetic materials used in estimating genetic gain.

Pipeline	No. of test entries	No. of checks	Total entries	Year of origin	Year of testing
Short duration (SD)	260	8	268	2006–2019	2010–2022
Medium duration (MD)	281	9	290	2004–2019	2010–2022
Late duration (LD)	239	12	251	2006–2018	2010–2022

### 2.2 Field design and data collection

Seventeen environments were used to evaluate SD cowpea genotypes. There were 13 and seven environments for MD and LD pipelines, respectively. The geographical information for all the locations has been summarized in Table 2. The test sites spanned a range of agroecologies in the West African region. These included the Sahel, Sudan savanna, Guinea savanna, and rainforest‐savanna transition zone. Different numbers of lines were evaluated each year, ranging from 10 to 28 lines for SD, 20 to 50 for MD, and 10 to 39 for LD pipelines. All trials were established using an alpha lattice design with three replications. Each plot had four rows, planted at a spacing of 0.75 m between rows and 0.2 m within the row. Grain yield and 100‐seed weight (Hsdwt) were recorded from the two central rows of each plot after harvest and threshing.

**TABLE 2:** Descriptions of sites used to evaluate cowpea lines for the genetic gain estimation.

Location	Country	Coordinates	Agroecology
Minjibir	Nigeria	12°8.73′N, 8° 39.97′E	Sudan savanna
Bayero University Kano (BUK)	Nigeria	11.9645° N, 8.4309° E	Sudan savanna
Bauchi	Nigeria	10.2801° N, 9.7945° E	Sudan savanna
Shika	Nigeria	11° 15′N, 7° 32′E	Northern Guinea savanna
Malamadori	Nigeria	12.5510° N, 10.0276° E	Sahel
Ibadan	Nigeria	7.4970° N, 3.9071° E	Forest savanna transition
Toumnia	Niger	13°28.7′N, 9°0.78′E	Sahel
Zakpota	Benin	7.2169° N, 2.1843° E	Southern Guinea savanna
Cinzana	Mali	12° 15′ N, 5° 57′ W	Sahel
Djalingo	Cameroon	9.2283° N, 13.4449° E	Sudan savanna
Guiring	Cameroon	10°37'27.1′, 014°22′13.7′	Sahel savanna
Magaria	Niger	13.0035° N, 8.9080° E	Sahel
Maradi	Niger	13.5010° N, 7.1036° E	Sahel
Samaru	Nigeria	11.1623° N, 7.6290° E	Northern Guinea savanna

### 2.3 Statistical analysis

Realized genetic gain was estimated following the method described by Mackay et al. (2011) using the historical data collected from the IITA cowpea breeding program's multi‐environment trials (METs) covering three distinct cowpea breeding pipelines, SD, MD, and LD. We used “historical information method” for the estimation of realized genetic gain as opposed to “era trial method” to exploit the existing historical data in the breeding program (Covarrubias‐Pazaran, 2020).

Individual‐environment analyses were first conducted for each trait to estimate genetic variance components and single‐environment broad‐sense heritability (*H* ^2^_se_), as a measure of repeatability. A linear mixed model with random genotypes was used (Cullis et al., 2006):

(1) $$Y_{ijk}=\mu +g_{i}+r_{j}+\;b{\left(r\right)}_{jk}+\varepsilon_{ijk}$$

where $Y_{ijk}$ denotes the observed value of trait for the *i*th genotype in the *k*th block within the *j*th replicate, $g_{i}$ is the random effect of the *i*th genotype, $r_{j}$ is the effect of the *j*th replication, $b{\left(r\right)}_{jk}$ is the effect of *k*th incomplete block nested within the *j*th replication, and $\varepsilon_{ijk}\;$ is the random residual effect associated with the observation of the *i*th genotype in the *kth* incomplete block within the *j*th replicate, $∼N(0,\sigma_{e}^{2})$.

We computed *H* ^2^_se_, the single‐environment broad‐sense heritability (repeatability), using the formula described in Cullis et al. (2006):

(2) $$H_{\mathrm{se}}^{2}=1-\frac{V_{\Delta }^{\mathrm{BLUP}}}{2\sigma_{g}^{2}}$$

where $V_{\Delta }^{\mathrm{BLUP}}$ is the average standard error of the best linear unbiased predictions (BLUP) and $\sigma_{g}^{2}$ is the genotypic variance. The estimate of $H_{\mathrm{se}}^{2}$ provided a measure of experimental repeatability and/or data quality.

Then we adopted a two‐stage approach following Smith et al. (2001) to estimate the performances of the genotypes across environments. In the first stage, we used the same linear mixed model (Equation 1) but considered genotype a fixed effect to estimate the BLUE (best linear unbiased estimates) and their standard errors for each genotype. In the second stage, we fit the model below (Mackay et al., 2011):

(3) $$y_{ijk}=\mu +G_{i}+Y_{j}+GY_{ij}+S_{jk}+e_{ijk}$$

where $y_{ijk}$ is the vector of BLUE of the *i*th genotype in year *j* at site *k* derived from the linear mixed models (Equation 1), μ is the overall mean, $G_{i}$ is the effect of the *i*th genotype, $Y_{j}$ is the effect of the *j*th year, $GY_{ij}$ is the interaction of genotype *i* in year *j*, $S_{jk}$ is the effect of location *k* within year *j*, and $e_{ijk}$ is the residual, due to the combined effects of within‐trial error and genotype × location within‐year interaction. We considered genotype as a fixed effect and used a weighted‐combined mixed model that included the genotype BLUE and their corresponding weights. These weights were calculated as the inverse of the squared standard errors of the BLUE of the genotypes from the initial stage analysis (Smith et al., 2001). We also included year as a fixed effect to separate the effects of agronomic practices or climate changes from genetic factors (Mackay et al., 2011). This enabled us to calculate adjusted genotype means across environments. Using a mixed model with random effects for genotype and year could lead to inaccurate results because of expected and potentially nonlinear trends over time for both genotype and year effects (Mackay et al., 2011). The dispersion of genotypes based on BLUE was visualized using violin plots generated in ggplot2 (Wickham, 2016).

Finally, we studied trends (realized genetic gain) using weighted regression analysis of the estimated genotypes adjusted means (BLUE) on year of origin. The form of the linear regression model used is as shown:

(4) $$y_{i}=\beta_{0}+\beta_{1}X_{i}+\varepsilon_{i}$$

where $y_{i}$ is the *i*th genotype adjusted mean (BLUE) of each trait, $X_{i}$ is the year of origin for genotype, $\beta_{0}$ is the regression intercept, and $\beta_{1}$ is the slope of the regression, which is equivalent to the absolute rate of realized genetic gain per year. The weights were the inverse of the squared standard errors of the adjusted means of the genotypes. Percentage change in realized genetic gain (%ΔRG) for each pipeline was then calculated as the ratio of the regression's slope ($\beta_{1}$) to the *y*‐intercept of the regression ($\beta_{0}$) plus the slope multiplied by the first year of origin ($X_{i}$) as shown:

(5) $$\%\Delta \mathrm{RG}=\left[\frac{\beta_{1}}{\beta_{0}\;+\;\beta_{1}X_{0}}\right]\times 100.$$

Predicted genetic gain (*∆G*) was estimated based on the MET data for year 2022 of genetic materials evaluated in AYT, the stage where recycling usually occurs. Inflated estimates of genetic variance occur when variance components are estimated from single‐environment analysis because of the confounding effect of genotype‐by‐environment interaction effect (Yan, 2014). Therefore, a combined‐environment analysis was conducted for each trait to calculate unbiased heritability estimates. The mixed model that was used for variance component estimates and MET broad‐sense heritability (*H* ^2^_met_) is given as follows:

(6) $$Y_{ijkl}=\mu +g_{i}+e_{l}+r{\left(e\right)}_{jl}+b{\left(\mathrm{er}\right)}_{ljk}+ge_{il}+\varepsilon_{ijkl}$$

where $Y_{ijkl}$ is the observed phenotypic value of trait for the *i*th genotype in the *l*th location and *j*th replication, μ is the overall mean, $g_{i}$ is the random effect of the *i*th genotypes, $e_{l}$ is the random effect of *l*th location, $r{\left(e\right)}_{jl}$ is the effect of *j*th replication nested within *l*th location, $b{\left(er\right)}_{ljk}$ is the effect of *k*th block nested within *j*th replication and *l*th location, $ge_{il}$ is the random effect of genotype‐by‐location interaction, and $\varepsilon_{ijkl}$ is the residual variance. The *H* ^2^_met_ used in the predicted genetic gain computation was estimated based on the following expressions (Yan, 2014):

(7) $$H_{\mathrm{met}}^{2}=\frac{\sigma_{g}^{2}}{\sigma_{g}^{2}\;+\;\frac{\sigma_{\mathrm{ge}}^{2}}{l}\;+\;\frac{\sigma_{\varepsilon }^{2}}{lr}}$$

where $\sigma_{g}^{2}$ and $\sigma_{\varepsilon }^{2}$ are the genotype and error variance components, respectively, $\sigma_{\mathrm{ge}}^{2}$ is the variance component for genotype‐by‐location interaction, and l and r are the number of locations and replications, respectively. The predicted genetic gain (*∆G*) was then calculated using the Breeder's equation:

(8) $$\Delta G=\frac{i\;\times \;\sigma_{g\;}\;\times r}{L}$$

where i is the selection intensity estimated as the mean of the deviations from the population mean, measured in units of the phenotypic standard deviation of the population (Mackay, 2020), $\sigma_{g}$ is the genetic variance, r is the accuracy (the square root of the *H* ^2^_met_), and L is the recycling time, which was 4 years from hybridization to AYT. The same traits as for the realized genetic gain were used for the calculation. All the genetic analyses were conducted on the historical cowpea data in R software (R Core Team, 2023), using ASReml package (The VSNi Team, 2023).

## 3 RESULTS

### 3.1 Performance of tested genotypes

**FIGURE 1:**
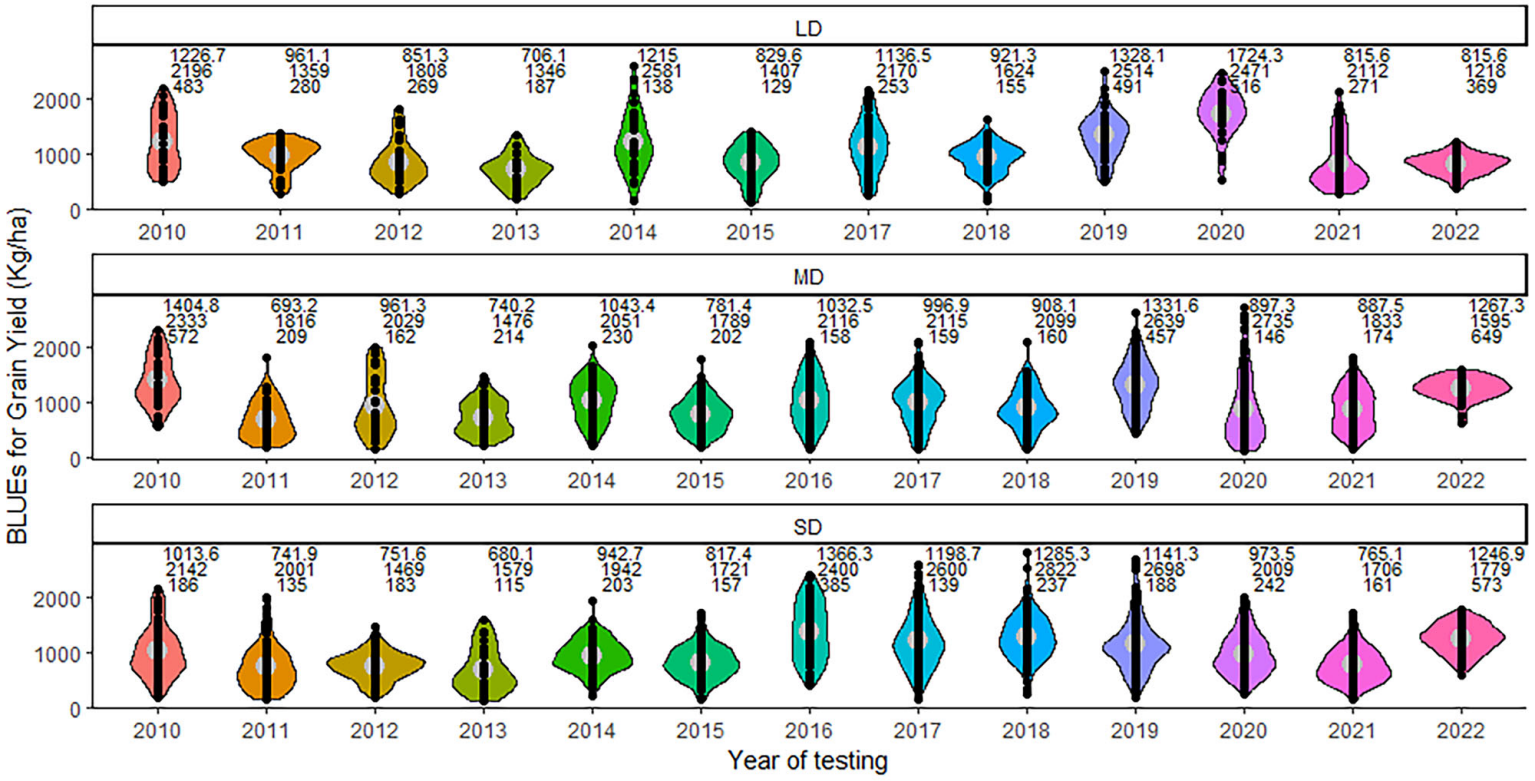
Distribution of grain yield by year of genotypes testing. Values printed on top of each violin plot are summary statistics presented in sequence, starting with mean, followed by maximum and minimum. The values plotted are best linear unbiased estimates (BLUE) from individual locations in a particular year; hence, the range within a year indicates location differences. The panels labeled LD, MD, and SD refer to late duration, medium duration, and short duration breeding pipelines, respectively. The means are also represented by the gray point inside the violin plots.

BLUE of individual environments were used to examine genotype performance in each testing year, and, therefore, yearly dispersion reflected the effect of environments on productivity. The distribution of grain yield based on BLUE of genotypes per year of testing in the three pipelines has been presented in Figure 1. The grain yield performance of test genotypes in the SD pipeline was the lowest in year 2013, and it ranged from 115 to 1579 kg/ha, with a mean of 680 kg/ha, while the highest performances were obtained in years 2016 (range: 385—2800 kg/ha, mean: 1366 kg/ha), 2018 (range: 237—2822 kg/ha, mean: 1285 kg/ha), and 2022 (range: 573—1779 kg/ha, mean: 1247 kg/ha). Yield performance in the MD pipeline was the lowest in 2011 (range: 209—1816 kg/ha, mean: 693 kg/ha), while the highest grain yields were obtained in years 2010 (range: 572—2333 kg/ha, mean: 1405 kg/ha), 2019 (range: 457—2639 kg/ha, mean: 1332 kg/ha), and 2022 (range: 649—1595 kg/ha, mean: 1267 kg/ha). The LD pipeline depicted the year 2013 as having the lowest grain yield performance, with a range of 187—1346 kg/ha and a mean of 706 kg/ha, while high performances were registered in years 2020 (range: 516—2471 kg/ha, mean: 1724 kg/ha), 2019 (range: 491—2514 kg/ha, mean: 1328 kg/ha), and 2010 (range: 483—2196 kg/ha, mean: 1227 kg/ha). Overall, a comparison among means of test years depicted grain yield to vary from 680.1 to 1366.3 kg/ha for SD pipeline, 693.2 to 1404.8 kg/ha for MD pipeline, and 706.1 to 1724.3 kg/ha for LD pipeline (Figure 1).

**FIGURE 2:**
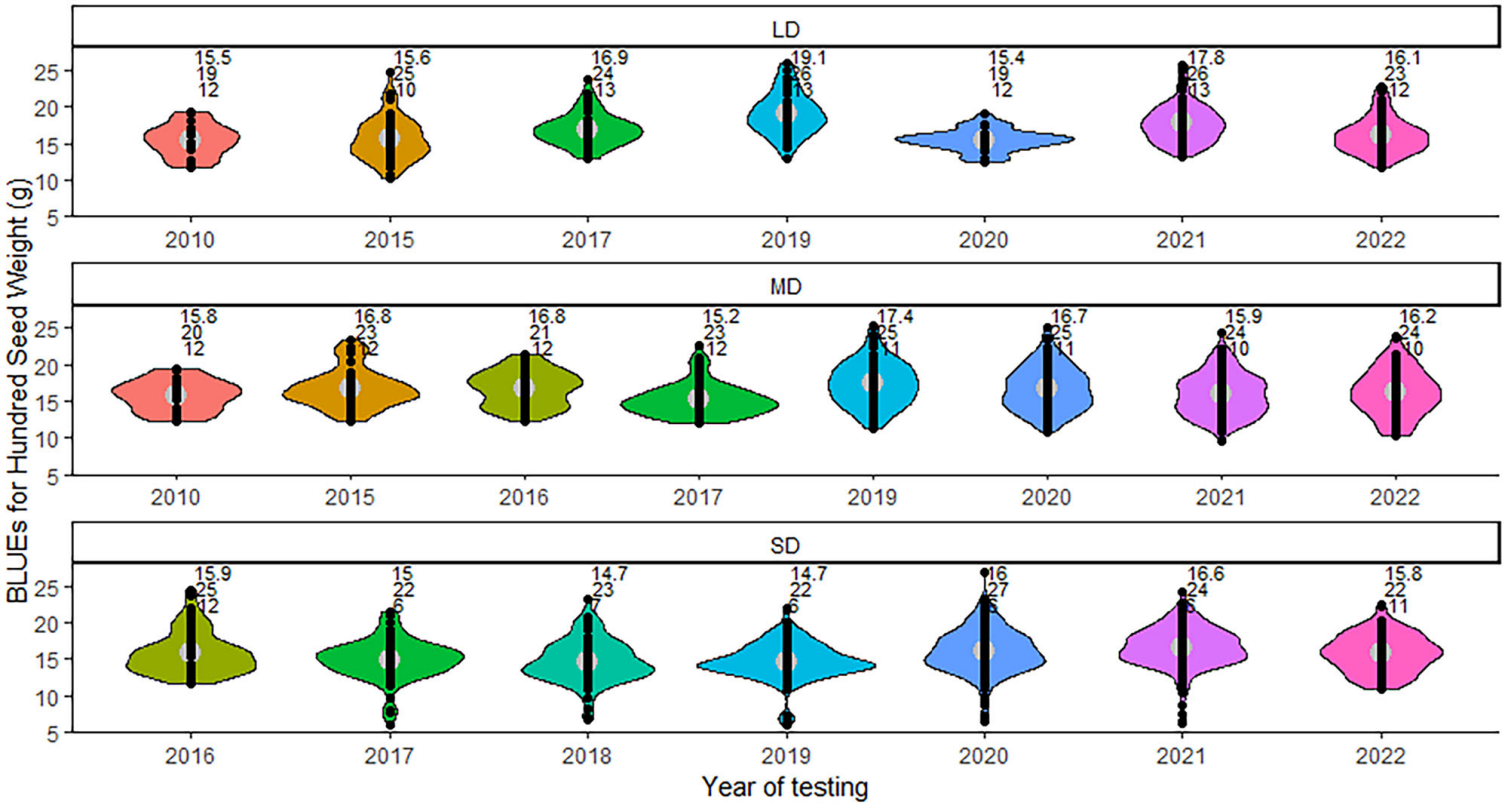
Distribution of 100‐seed weight by year of genotype testing. Values printed on top of each violin plot are summary statistics presented in sequence, starting with the mean, followed by the maximum and minimum. The values plotted are best linear unbiased estimates (BLUE) from individual locations in a particular year; hence, the range within a year indicates location differences. The panels labeled LD, MD, and SD refer to late duration, medium duration, and short duration breeding pipelines, respectively. The means are also represented by the gray point inside the violin plots.

Similarly, the distribution of genotype performance using the BLUE for Hsdwt in each year of testing has been presented in Figure 2. The SD pipeline had relatively low Hsdwt values in 2019 and 2018, with ranges of 6—22 g and 7—23 g, respectively, each having a mean of 15 g. However, Hsdwt was relatively high in 2021 (range: 6—24 g, mean: 17 g) and 2020 (range: 6—27 g, mean: 16 g). In the MD pipeline, the year 2017 had the lowest Hsdwt, with the values varying from 12 to 23 g and a mean of 15 g, while the year 2019 registered the highest value, with a range of 11—25 g and a mean of 17 g. On the other hand, the LD pipeline had low performance in 2020, with Hsdwt ranging from 12 to 19 g and a mean of 15 g, while the highest performance in this pipeline was registered in 2019, with values varying from 13 to 26 g, and a mean of 19 g. Overall, when the means of test years were compared, the numerical differences ranged from 14.7 to 16.6 g for the SD pipeline, 15.2 to 17.4 g for the MD pipeline, and 15.4 to 17.8 g for the LD pipeline (Figure 2).

In addition, we examined heritability for each test environment to ascertain the repeatability of results. The range of heritability values for environments in each year of testing has been presented in Table 3. Low to high single environment heritability estimates were obtained for grain yield and Hsdwt. In the SD pipeline, environments with heritability estimates below 20% were obtained in 2013 and 2021 for grain yield and 2016 for Hsdwt. Similarly, the MD pipeline recorded heritability estimates of less than 20% in 2011, 2016, and 2022 for grain yield and in 2016 and 2017 for Hsdwt (Table 3). The LD pipeline, on the other hand, depicted heritability for grain yield to be above 20% in all years, while it was only in 2019 where Hsdwt scored below the stated threshold (Table 3). Based on these results, we excluded 18%, 30%, and 14% of environments in the SD, MD, and LD pipelines, respectively, from the genetic gain estimation as part of data quality control, given that these environments had revealed low repeatability of results.

**TABLE 3:** Range of single environment heritability estimates in each year of genotype testing.

Short duration					Medium duration				Late duration			
	Grain yield		Hsdwt		Grain yield		Hsdwt		Grain yield		Hsdwt	
Year	*H* ^2^_se_	*N*	*H* ^2^_se_	*N*	*H* ^2^_se_	*N*	*H* ^2^_se_	*N*	*H* ^2^_se_	*N*	*H* ^2^_ae_	*N*
2010	0.34–0.89	7	–	–	0.22–0.83	2	0.91	1	0.32–0.58	2	0.85	1
2011	0.36–0.86	5	–	–	0.18–0.74	4	–	–	0.54–0.86	2	–	–
2012	0.52–0.69	5	–	–	0.12–0.94	4	–	–	0.64–0.65	2	–	–
2013	0.02–0.63	4	–	–	0.12–0.84	4	–	–	0.54–0.9	3	–	–
2014	0.29–0.87	6	–	–	0.23–0.81	4	–	–	0.72–0.79	3	–	–
2015	0.88–0.98	6	–	–	0.26–0.91	5	0.85–0.93	2	0.59–0.95	4	0.42–0.95	3
2016	0.59–0.66	3	0.15–0.96	3	0.07–0.66	5	0.00–0.96	3	–	–	–	–
2017	0.56–0.88	5	0.81–0.96	3	0.40–0.98	6	0.02–0.89	2	0.37–0.78	3	0.59–0.85	2
2018	0.63–0.9	3	0.89–0.93	3	0.84–0.85	2	–	–	0.64–0.84	2	–	–
2019	0.37–0.81	5	0.89–0.98	5	0.61–0.9	4	0.72–0.95	4	0.37–0.68	3	0.00–0.93	3
2020	0.20–0.87	7	0.71–0.95	7	0.23–0.7	5	0.3–0.98	4	0.67	1	0.63	1
2021	0.11–0.68	4	0.68–0.89	5	0.49–0.72	4	0.54–0.9	4	0.35–0.68	3	0.3–0.92	5
2022	0.36–0.66	2	0.77–0.92	2	0.17–0.54	2	0.82–0.97	2	0.75–0.77	2	0.91–0.93	2

*Note*: *N* is the number of environments in each year, Hsdwt refers to 100‐seed weight (g), *H* ^2^_se_ is the single‐environment broad‐sense heritability presented as a range of values for the environments in each year of testing, and the symbol “–”indicates the absence of data record.

### 3.2 Trial connectivity

Since each year had independent trials with a unique set of genetic materials in each of the three breeding pipelines, we examined connectivity across trials before the estimates of genetic gain. The results of trial connectivity are presented in Table 4. In the SD pipeline, 67 trials were conducted across 17 environments. In this pipeline, 268 entries were involved; out of these, 260 were test entries, while 8 were common checks across trials.

**TABLE 4:** Pipeline entries tested per year and connectivity across years from 2010 to 2022.

	Short duration												
	2010	2011	2012	2013	2014	2015	2016	2017	2018	2019	2020	2021	2022
2010	26	12	7	5	5	2	0	1	1	1	1	1	0
2011		20	9	5	5	2	0	1	1	1	1	1	0
2012			15	7	7	3	0	1	1	1	1	1	0
2013				10	10	4	0	1	1	1	1	1	0
2014					18	6	0	1	1	1	1	1	0
2015						20	0	1	1	1	1	1	0
2016							30	0	1	1	3	1	1
2017								30	5	2	1	3	2
2018									28	7	4	4	3
2019										28	7	4	3
2020											35	5	3
2021												43	4
2022													45

	Medium duration												
	2010	2011	2012	2013	2014	2015	2016	2017	2018	2019	2020	2021	2022
2010	25	12	8	8	3	3	1	2	2	2	2	1	3
2011		20	10	10	4	4	1	2	2	2	2	2	3
2012			20	20	8	8	1	2	2	2	2	1	3
2013				28	14	14	1	2	2	2	2	1	3
2014					24	24	1	2	2	1	1	1	2
2015						39	1	2	2	1	1	1	2
2016							35	3	2	1	1	2	2
2017								31	3	1	1	1	3
2018									25	5	3	1	3
2019										27	11	1	3
2020											35	2	3
2021												50	1
2022													45

	Late duration												
	2010	2011	2012	2013	2014	2015	2016	2017	2018	2019	2020	2021	2022
2010	20	12	4	4	3	2	–	1	1	1	1	1	1
2011		25	6	6	4	2	–	1	1	1	1	1	1
2012			10	10	6	2	–	1	1	1	1	1	1
2013				14	10	3	–	1	1	1	1	1	1
2014					12	3	–	1	1	1	1	1	1
2015						20	–	1	1	1	1	1	1
2016							–	–	–	–	–	–	–
2017								32	3	1	1	1	1
2018									35	1	1	1	2
2019										27	3	2	4
2020											32	5	2
2021												39	1
2022													45

*Note*: On the diagonals are the number of entries tested each year, above the diagonal is the number of common entries shared between years of testing, and the symbol “–”indicates an absence of a data record.

The MD pipeline consisted of 53 trials conducted across 13 environments. In this pipeline, 290 entries were involved; out of these, 281 were test entries, while 9 were checks. For the LD pipeline, 35 trials were conducted across seven environments. A total of 251 entries were evaluated, 239 being the test entries, while 12 were check genotypes. For each of the three pipelines, the number of entries varied yearly, and connectivity was provided by having at least one common check in all the years of testing, except between the year 2022 and the years 2010–2015 for the SD pipeline.

### 3.3 Realized genetic gain

**FIGURE 3:**
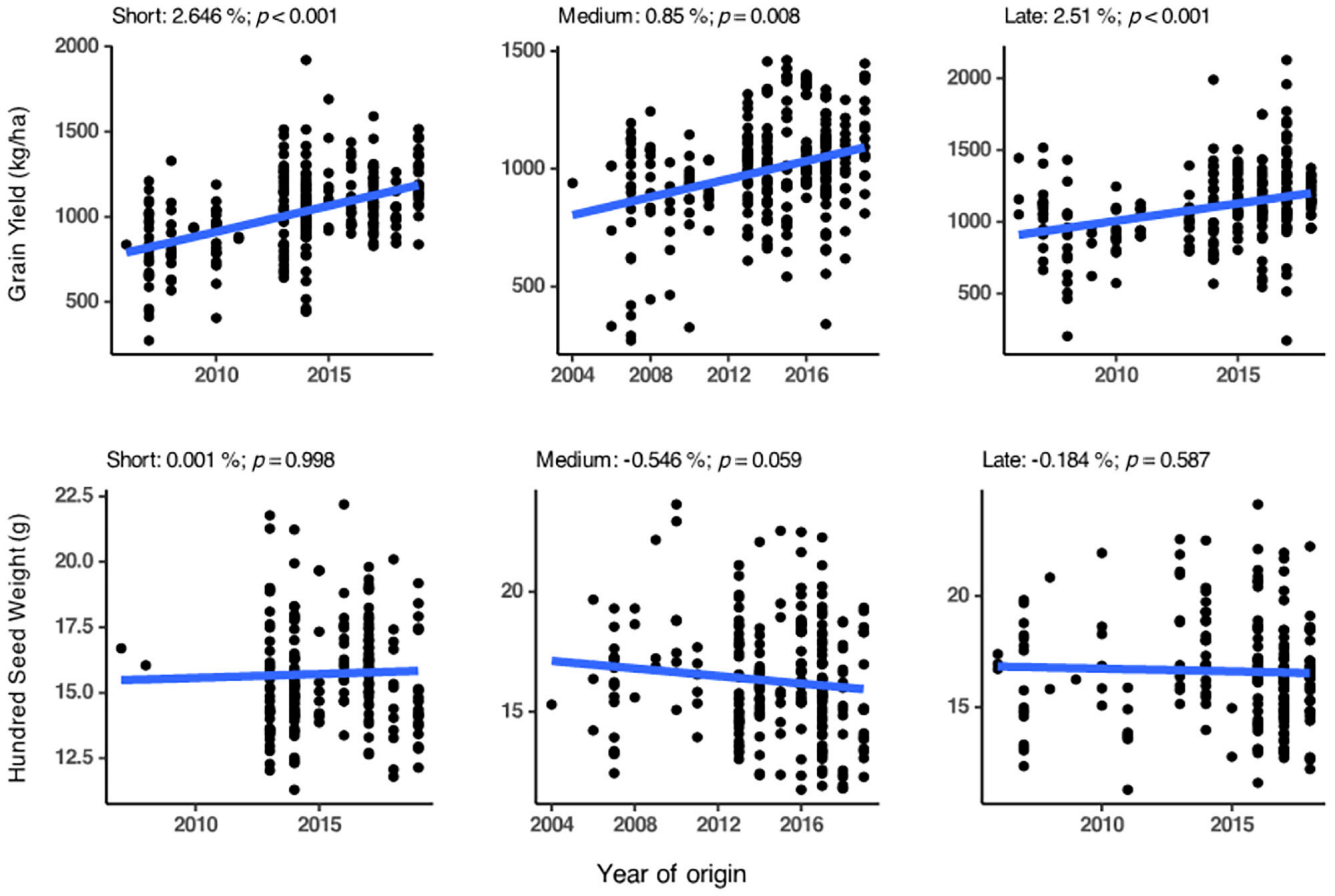
Regressions of grain yield and 100‐seed weight against year of origin for the short, medium, and late‐duration cowpea breeding pipelines.

Realized genetic gain was conducted for the three breeding pipelines. Regression analysis detected a significant positive association between grain yield and year of origin in all three pipelines (Figure 3). The slopes of grain yield versus year of origin were significantly greater than zero. Grain yield has increased linearly across the three breeding pipelines since the year when the first sets of cowpea lines were developed, in 2006 for the SD and LD pipelines and 2004 for the MD. The absolute realized genetic gain for grain yield in the SD pipeline was 22.75 kg/ha/year, which translates to a rate of 2.65% (Figure 3). The MD pipeline registered the realized genetic gain in yield of 7.91 kg/ha/year, equivalent to 0.85% gain per year (Figure 3), while the LD had a gain in yield of 22.82 kg/ha/year, translating to 2.51% (Figure 3). The regression between Hsdwt and year of origin was not statistically significant for all three breeding pipelines, indicating that no gain was observed.

### 3.4 Predicted genetic gain

MET data for the year 2022 was used to compute predicted genetic gain by leveraging genetic variance and heritability estimates in the Breeder's equation (Table 5). Genetic variances were high for all three pipelines, suggesting sufficient genetic diversity to warrant effective selection in future populations derived from these genotypes. Consequently, heritability estimates were high for grain yield and Hsdwt in all three breeding pipelines. The heritability estimates ranged from 0.55 for grain yield in SD to 0.86 for LD pipelines. For Hsdwt, heritability estimates varied from 0.94 in SD to 0.97 in LD. Predicted genetic gain reflecting the progress expected from recycling the best parents at the AYT stage in the year 2022 was different for each breeding pipeline. For instance, the predicted genetic gain in grain yield was 37.28 kg/ha per year for SD, 28.0 kg/ha for MD, and 34.85 kg/ha for LD pipelines (Table 5). In the case of Hsdwt, the predicted genetic gain was 0.48 g/year in the SD pipeline, 0.65 g/year in MD, and 0.55 g/year in LD (Table 5).

**TABLE 5:** Estimates of variances, heritabilities, and predicted genetic gain for grain yield and 100‐seed weight for short‐, medium‐, and late‐duration cowpea breeding pipelines in 2022.

Pipeline	Parameter	GY (kg/ha)	Hsdwt (g)
Short	i	1.04	1.04
	*H* ^2^_met_	0.7	0.94
	$\sigma_{g}^{2}$	29,016.15	3.55
	ΔG	37.28	0.48
Medium	i	1.01	1.01
	*H* ^2^_met_	0.55	0.97
	$\sigma_{g}^{2}$	22,314.12	7.57
	ΔG	28	0.68
Late	i	1.00	1.00
	*H* ^2^_met_	0.86	0.96
	$\sigma_{g}^{2}$	23,003.82	5.05
	ΔG	34.85	0.55

*Note*: The recycling time is 4 years, and 15 genotypes are recycled at the AYT stage. GY is the grain yield, Hsdwt is 100‐seed weight, *H* ^2^_met_ is the multi‐environment broad‐sense heritability, $\sigma_{g}^{2}$ is the genetic variance, ΔG is the predicted genetic gain, and i is the selection intensity.

## 4 DISCUSSION

To develop crop varieties that meet the growing demand for food and feed, in addition to various industrial uses, breeders are confronted with the need to continuously increase genetic gain at even higher rates while they close the gaps that remain between the yield potential in breeders’ trials and the actual yield in farmers’ fields (Xu et al., 2017). It is estimated that breeding programs in the developing world will need to deliver elevated rates of genetic gain to grapple with the 21^st^‐century challenges of more than 50% greater demands for food products, climate change, and natural resource limitations (Genetic Gains Working Group, 2016). Without a systematic assessment, it is difficult to gauge genetic progress in a breeding program to be able to activate changes that can enhance a positive trend. Genetic progress measured through changes in trait performance over time is important in determining the effectiveness of breeding programs (Kumar et al., 2021). This is because breeding programs often generate new test genotypes yearly, motivated by the assumption that the new genotypes will surpass the older ones based on improved performance and the inclusion of desirable features that support farmer productivity and risk mitigation.

### 4.1 Genetic gain realized in the past 12–15 years

Since the inception of a cowpea breeding program at IITA in 1970, there has been considerable progress ranging from assembly and characterization of over 15,000 germplasm, development of breeding lines, and release of varieties with improved agronomic traits and farmer preferred attributes (Boukar et al., 2011, 2019; C. A. Fatokun et al., 2012; Ongom, Fatokun, Togola, Oyebode, et al., 2021; S. R. Singh et al., 1989; B. B. Singh et al., 1997; Togola et al., 2020). The varieties developed by IITA have been released globally in more than 70 countries, and these have continued to make significant contributions to the food needs of millions of communities, especially in the tropics and subtropical regions where cowpea is considered a major food security and nutritional crop.

The genetic gain made in the cowpea breeding program was assessed in three key breeding pipelines: SD from 2006 to 2019, MD from 2004 to 2019, and LD from 2006 to 2018, using historical AYT MET data from 2010 to 2022. In this approach to realized genetic gain estimation, not all the genotypes were evaluated together in an experiment as is commonly done for era trials (Covarrubias‐Pazaran, 2020), rather, historical phenotypic data from time‐representative samples of genotypes in AYT were evaluated across many years as the program progresses. The fact that each year of evaluation had new sets of genotypes introduces the challenge of connectivity among time‐window entries, which could bias the estimate of realized genetic gain (Covarrubias‐Pazaran, 2020). According to Rutkoski (2019a), the estimates of realized genetic gain require good connectivity between years, and the use of long‐term checks can help increase connectivity. In the present study, connectivity was resolved using common checks included in the trials. For all three breeding pipelines, at least one check variety was common across years, except for that between 2022 and the years 2010—2015. Model comparisons revealed that in the event of poor connectivity, the use of pedigree or marker to estimate the breeding values would be recommended, and in that case, data would be connected by genetic relationships that exist in the breeding materials (Covarrubias‐Pazaran, 2020; Garrick, 2010; Seck et al., 2023).

Genetic gain assessment of grain yield in the present study revealed that efforts have been successful in increasing yield in the three key cowpea breeding pipelines. The realized genetic gain of 22.75 kg/ha/year (2.65%), 7.91 kg/ha/year (0.85%), and 22.82 kg/ha/year (2.51%) for SD, MD, and LD breeding pipelines demonstrates the success of our breeding strategy implemented during the specified time spans in each pipeline. The results indicate that more progress was achieved in the SD and LD breeding pipelines than in the MD pipeline. An assessment of genetic gain in cowpea was last conducted in 2011 following the era trial approach, in which 15 determinate and 16 semi‐determinate breeding lines from 1997 to 2004 were evaluated together for 2 years in a single location (Kamara et al., 2011). The genetic gain assessment was not pipeline‐specific; however, the authors reported an annual gain of 2.93% and 4.4% in the yield of determinate and semi‐determinate cowpea varieties, respectively (Kamara et al., 2011). The observed difference is not surprising, given that the measured rate of genetic gain highly depends on the productivity of the test environments in which that gain is estimated (Boehm et al., 2019; Rincker et al., 2014). The present study used historical data accumulated from 17 test environments across multiple agroecologies (Table 2), which depicts a representation of genetic gain realized in the West and Central African regions. In common bean (*Phaseolus vulgaris* L.), the genetic gain for black‐grained varieties developed by the Brazilian Agricultural Research Enterprise (EMBRAPA) breeding program was estimated at an annual rate of 1.23%, covering a period of 16 years. In soybean, an estimate of genetic gain for a period of 80 years depicted an average annual seed yield gain of 0.88% (17.6 kg/ha/year), 0.68% (13.5 kg/ha/year), and 0.52% (10.3 kg/ha/year) for three respective maturity groups (Boehm et al., 2019). The CIMMYT (International Maize and Wheat Improvement Center) maize breeding program reported a genetic gain of 3.39% for grain yield under managed waterlogging stress and 1% under optimal conditions (Prasanna et al., 2022). The current genetic gain estimates for cowpea show significant progress compared to other legumes, indicating that a yield plateau has not yet been reached. This gain resulted in part from breeding modernization efforts adopted in the past years. These included the adoption of SSD for rapid generation advancement, which helped to reduce the parent recycling period from 6 to 4 years, the use of molecular markers for parental fingerprint and hybridity authentication as part of quality control (Ongom, Fatokun, Togola, Salvo, et al., 2021), and the adoption of mechanization including mechanized trial planting and threshing. The program also adopted digital data capture using tablets and data management using breeding management system, coupled with the application of more efficient experimental designs and robust statistical analysis including the use of mixed linear models that allows selection based on BLUP.

### 4.2 Genetic gain projection

To gauge the breeding program's projected progress, we conducted the predicted genetic gain based on the 2022 AYT data from multiple locations. Predicted genetic gain, unlike realized genetic gain, utilizes the components of the Breeders’ equation calculated for a single season to estimate the response to selection and infer the rate of genetic gain (Burrows, 1972; Falconer & Mackay, 1996; Walsh & Lynch, 2018). This estimate is dependent on measurements of genetic variance, heritability, and length of the breeding cycle. Our study revealed moderate to high genetic variance, reflected in the heritability estimates ranging from 55% to 86% for grain yield and 94% to 97% for Hsdwt. Similar ranges of heritability have been reported for cowpea grain yield and Hsdwt (Nwosu et al., 2013; Ongom, Fatokun, Togola, Oyebode, et al., 2021; Owusu et al., 2021). Predicted gain in yield of 37.28, 28.0, and 34.85 kg/ha/year and gain in Hsdwt of 0.48, 0.65, and 0.55 g/year for SD, MD, and LD pipelines, respectively, suggested that parental lines recycled at this stage of the breeding program across SD, MD, and LD genotypes had sufficient genetic variance; hence, effective selection in the derivative populations is possible. It is worth noting that the predicted genetic gain method only indicates the direction in which the program is moving rather than giving an accurate estimate of genetic gain (Covarrubias‐Pazaran, 2020). Continuous selection for key traits in a breeding program can compromise genetic diversity and may limit improvement. Breeders are therefore challenged to strike a balance, that is, increasing genetic gain while maintaining sufficient genetic diversity. Predicted genetic gain is useful in assessing residual genetic diversity in the population to allow effective selection. In the present case, the results demonstrated positive progress for both yield and Hsdwt.

### 4.3 Prospects of genomic integration

Genetic gain can be accelerated by reducing the breeding cycle and the recycling of parents having high breeding values in the breeding program. It has been shown that molecular integration into breeding programs is the key to achieving enhanced genetic gain (Biswas et al., 2023). In recent years, cowpea has seen significant advances in genomics, which is projected to have a positive impact on the rate of genetic gain. The latest advances include the development of the cowpea reference genome based on IITA variety IT97K‐499‐35 (Lonardi et al., 2019). Six additional cowpea genotypes have recently been assembled, together with the IT97K‐499‐35 genome, forming the pan‐genome of cowpea (Liang et al., 2022). Several genotyping platforms have also been developed, including the Illumina Cowpea iSelect Consortium Array, which has 51,128 single nucleotide polymorphisms (SNPs) (Muñoz‐Amatriaín et al., 2017), Illumina GoldenGate Assay with 1536 SNPs (Muchero, Diop, et al., 2009), DArTseq that combines diversity array technology with next generation sequencing, and the mid‐density genotyping panel having 2602 DArTag SNPs (Ongom et al., 2024). In addition, low‐density genotyping panels have been developed based on KASP (kompetitive allele‐specific polymerase chain reaction) technology. More genomic resources and tools utilized by the cowpea breeding program have been extensively reviewed (Abdoul et al., 2023; Boukar et al., 2018, 2016). These resources are being exploited for quantitative trait loci (QTL) mapping of key traits such as aphid resistance (Huynh et al., 2015; Kusi et al., 2018; Ongom et al., 2022), bacterial blight resistance (Agbicodo et al., 2010), heat tolerance (Lucas et al., 2013), drought tolerance (Muchero, Ehlers, et al., 2009), and striga resistance among others (Abdoul et al., 2023; Boukar et al., 2016). Among these QTL, aphid and bacterial blight resistance markers have been deployed in our program for forward breeding while striga, heat, drought, flower thrips, and seed size markers are being validated for utilization in marker‐assisted selection. Our breeding program also routinely deploys the low‐density KASP panel for quality control and assurance, especially for genetic purity, parental relatedness, and F_1_ hybridity assessments (Ongom, Fatokun, Togola, Salvo, et al., 2021). The genomic resources have also been used in the program to assess genetic diversity and population structure (Fatokun et al., 2018; Gbedevi et al., 2021; Ongom et al., 2024) and are also being used in the implementation of genomic selection in cowpea. These developments are expected to improve breeding efficiency and accelerate the rate of genetic gain in cowpea.

## 5 CONCLUSIONS

For a 12‐ to 15‐year period, that is, from 2006 to 2019, 2004 to 2019, and 2006 to 2018, when the three IITA cowpea breeding pipelines were evaluated, positive progress was made for realized genetic gains of grain yield, which was estimated at 2.65%, 0.85%, and 2.51%, respectively. However, no significant changes were detected for Hsdwt. The progress observed in grain yield resulted from past efforts in assembling many cowpea germplasms with sufficient genetic diversity, followed by effective selection methods. This was further reinforced by breeding modernization efforts that were adopted by the breeding program, which included improving field uniformity, using robust experimental designs like row‐column, use of mixed linear models in the analysis of trials to estimate BLUP, use of markers for quality control and assurance, and rapid generation advancement through SSD. Considering future breeding direction, predicted genetic gain in yield of 3.0% (SD), 2.2% (MD), and 4.4% (LD), and in Hsdwt of 3.0% (SD), 4.3% (MD), and 3.4% (LD) revealed that the cowpea breeding program at IITA is heading in the right direction. The predicted gain demonstrated that parents recycled at AYT from 2022 trials had sufficient genetic variation to warrant effective selection in the subsequent populations derived from these selected parents. Recent efforts have also initiated marker discovery and deployment including forward breeding for key traits, marker assisted backcrossing program, and genomic selection for complex traits, and these are expected to boost future rate of genetic gain in cowpea. Overall, the positive trend in cowpea yield improvement, coupled with the recent integration of genomics, suggests that there is a high chance to continue increasing the yield of cowpea, thereby generating hopes to meet the future food need.

## AUTHOR CONTRIBUTIONS

**Patrick Obia Ongom**: Conceptualization; data curation; formal analysis; investigation; methodology; software; validation; visualization; writing—original draft; writing—review and editing. **Christian Fatokun**: Investigation; resources; supervision; writing—review and editing. **Abou Togola**: Data curation; investigation; writing—review and editing. **Ibnou Dieng**: Conceptualization; formal analysis; investigation; methodology; software; validation; visualization; writing—review and editing. **Stella Salvo**: Conceptualization; resources; writing—review and editing. **Brian Gardunia**: Conceptualization; resources; writing—review and editing. **Saba Baba Mohammed**: Writing—review and editing. **Ousmane Boukar**: Conceptualization; funding acquisition; investigation; methodology; project administration; resources; supervision; validation; writing—review and editing.

## CONFLICT OF INTEREST STATEMENT

The authors declare no conflicts of interest.

## Data Availability

Data generated in this study will be available on the IITA website.
